# Estrogen-Related Receptors in Breast Cancer and Prostate Cancer

**DOI:** 10.3389/fendo.2015.00083

**Published:** 2015-05-26

**Authors:** Aya Misawa, Satoshi Inoue

**Affiliations:** ^1^Department of Anti-Aging Medicine, Graduate School of Medicine, The University of Tokyo, Tokyo, Japan; ^2^Division of Gene Regulation and Signal Transduction, Research Center for Genomic Medicine, Saitama Medical University, Saitama, Japan

**Keywords:** estrogen-related receptors, estrogen receptors, estrogen, androgen, breast cancer, prostate cancer

## Abstract

Estrogen-signaling pathways are implicated in the development of breast cancer and prostate cancer. Various studies have focused on additional signaling pathways, mediated by estrogen-related receptors (ERRs). ERRs are constitutively active receptors that share a high degree of homology with the classical estrogen receptors (ERs). However, they do not bind to estrogen, while ERs do. ERRs are involved in the development of alternative pathways that lead to the development of cancer and are regarded as potential therapeutic targets for the treatment of breast cancer and prostate cancer that do not respond to conventional therapies. In this review, we first present general structural features of ERRs. Then, we focus on breast cancer and prostate cancer, which are primarily hormone-dependent cancers, and summarizes recent progress in elucidating the involvement of each ERR in these two types of malignancies.

## Introduction

Among the 48 members of the nuclear receptor superfamily, the estrogen receptor (ER)-like subfamily (NR3) is one of the seven subfamilies and is composed of three groups: ERs(NR3A), estrogen-related receptors (ERRs or NR3B), and 3-ketosteroid receptors (NR3C), which include the androgen receptor (AR), progesterone receptor (PR), glucocorticoid receptor (GR), and mineralocorticoid receptor (MR). ERRs were initially thought to share a common biological function with ERs, but unexpectedly, they do not bind to estrogen or endogenous ER ligands and are considered orphan nuclear receptors (1).

Estrogens are natural hormones considered to play a major role in promoting the proliferation of both the normal and neoplastic breast epithelium. Growing evidences suggest that estrogen-signaling pathways are implicated in not only in the development of breast cancer but also in that of prostate cancer, which are both hormone-dependent cancers (2). ER-positive breast cancers are preferentially treated with antiestrogens such as selective ER modulators known as SERMs (including tamoxifen which is the most commonly used) or selective ER down-regulators known as SERDs (ICI 182,780) (3). Aromatase inhibitors are used in postmenopausal women to block the enzyme aromatase, which is involved in the final step of estrogen synthesis from circulating androgens (4). Androgen deprivation therapy (ADT) is used for the treatment of locally advanced, biochemically recurrent, and metastatic prostate cancer. However, after the initial response, these therapies for breast and prostate cancer fail and cancer will inevitably recur (5–7). The mechanisms underlying recurrence have not been fully clarified, but the acquisition of alternative intracellular ER signaling may be involved in hormonal therapy resistance.

**Figure 1:**
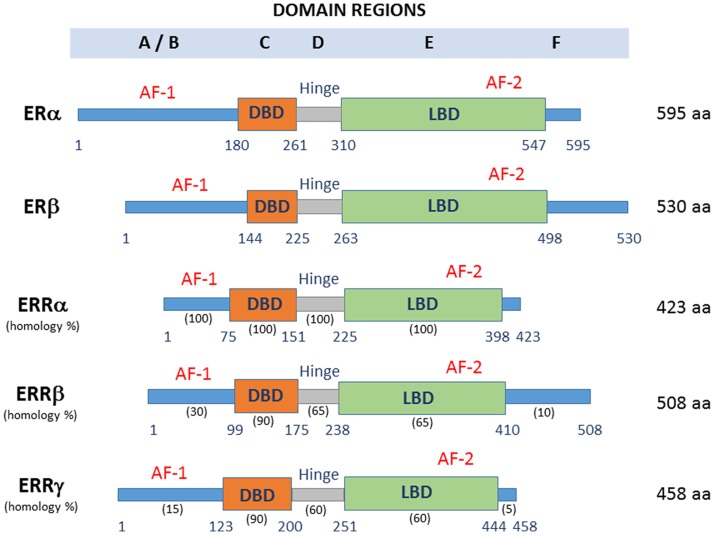
**Domain structure and protein length of ERRs and ERs**. DBD, DNA binding domain; LBD, ligand-binding domain; AF-1, activating function-1; AF-2, activating function-2. The percentage conservation between ERRα and the other two ERRs is shown for each of the five domains.

Various studies have focused on additional estrogen-related signaling pathways, mediated by ERRs (8–10). Sequence analysis reveals that ERRs and the classical ERs share a high degree of homology within their DNA and ligand-binding domains (LBDs) (11). The ERR family consists of three closely related members: ERRα, ERRβ, and ERRγ (Figure 1). ERRs, like other nuclear receptors, consist of six conserved regions (A–F): A/B domain containing the N-terminal domain (NTD), C domain with a DNA binding domain (DBD), D domain, which corresponds to the hinge region, E domain containing a putative LBD, and the F domain or C-terminal region. The transcriptional activation function-1 (AF-1) site is located in the N-terminal A/B domain, and its activity does not depend on the presence of activating ligands. The ligand-regulated activation function-2 (AF-2) site is located at E domain and it is known to synergize with AF-1 to regulate gene expression (12, 13). These receptors are constitutively active due to the structure of the LBD that leaves the AF-2 exposed and capable of binding coregulators in the absence of ligand binding.

## ERR Action Mechanisms

Like other nuclear receptors, the transcriptional activity of ERRs is dependent on the presence of coregulatory proteins, either corepressors or coactivators. Steroid receptor coactivator (SRC)-1, -2, and -3, which regulate hepatic metabolism, fat storage, and energy balance, have been shown to interact with one or more ERR isoform stimulating their transcriptional activity (14, 15). PPARγ coactivator (PGC)-1α and PGC-1β, which play essential roles in metabolic programs, have been shown to directly interact with ERRs, positively regulating the expression and activity or these nuclear receptors (16). The activity of the ERRs as transcription factors is generally inhibited through physical interaction with receptor interacting protein 140, RIP140, also known as nuclear receptor interacting protein 1, NRIP1, a corepressor that competes for interaction with PGC-1α for ERRs binding negatively regulating gene expression (17).

Estrogen-related receptors can bind to DNA on ERR response elements (ERREs) designated by the sequence 5′-TNAAGGTCA-3′ as well as the classical ER response elements (EREs) 5′-AGGTCANNNTGACCT-3′, where N represents any nucleotide (18–20). These orphan nuclear receptors play a central role in regulating cellular metabolism through the regulation of genes involved in glycolysis, oxidative phosphorylation, and tricarboxylic acid cycle (21). ERRα is present in tissues with high metabolism, such as the heart, kidney, intestinal tract, skeletal muscle, and brown adipose tissue (22). The expression of ERRβ and ERRγ is more restricted, being mainly expressed in the heart and kidney (22, 23). Both ERRα and ERRγ are upregulated in preadipocytes and pluripotent mesenchymal cells under adipogenic conditions, positively regulating lipid accumulation (24, 25). ERRβ and ERRγ are also expressed during the early embryonic development and in the central nervous system and spinal cord. Although ERRs have structural and functional similarities, mice deficient for each ERR exhibit different phenotypes, suggesting that these ERRs have specific and unique functions. ERRα-deficient mice are viable, but exhibit a phenotype characterized by reduced body weight, peripheral fat deposits, and resistance to high-fat diet-induced obesity (26). They also exhibit a loss of normal mitochondrial biogenesis (27). On the other hand, ERRβ-deficient mice are lethal due to impaired placenta formation (28). Interestingly, an important role of this orphan receptor in embryonic stem (ES) cells maintenance has been suggested, as it is able to regulate self-renewal in ES cells in cooperation with octamer-binding protein 4, Oct4, and sex determining region Y-box 2, Sox2, replacing kruppel-like factor 4, Klf4, and consequently enhancing the transcription of reprograming factors (29). ERRγ-deficient mice also die during the early postnatal period due to abnormal heart function (30). It has been reported that ERR overexpression increases glycolysis, while depletion results in the opposite effect, reducing aerobic glycolysis (31). Furthermore, ERRα and ERRγ were found to form heterodimers and bind to the promoters of the same target genes to regulate genes controlling both the glycolytic and the oxidative mitochondrial respiration phenotypes (32). These findings suggest that ERRs might contribute to a shift in cellular metabolism from normal mitochondrial oxidative phosphorylation to an aerobic glycolysis typical of cancer cells, known as the Warburg effect, increasing glucose consumption, and ATP production (33). However, a number of evidence suggest that ERRα and ERRγ have opposite functions in regulating cellular metabolism. ERRα could assist in setting a glycolytic profile required for the proliferation of rapidly dividing cells in normal tissues with high-energy demand and tumors consequently promoting cellular proliferation (34–39). By contrast, ERRγ high expression has been detected in tumors with better prognosis (36), and inhibition of this orphan receptor by a microRNA (miR-378*) induces a metabolic switch by promoting a Warburg-like phenotype (40). These findings illustrates the complexity of metabolic reprograming by ERRs in cancer cells, where ERRα and ERRγ could cooperate or play an opposite role in the metabolic transcriptional pathway depending on the cellular environment and the expression of distinct coregulators (41) (Table 1).

**Table 1:** **Expression of ERR isoforms and implication in breast cancer and prostate cancer**.

Receptor	Breast cancer			Prostate cancer		
	Expression	Implication	Reference	Expression	Implication	Reference
ERRα (NR3B1)	High	Transcriptional activator or repressor	(42)	High	Promotion of cell growth	(39)
		Promotion of cell growth and metastasis	(34, 39, 43, 44)	
ERRβ (NR3B2)	Uncertain	Uncertain	(36, 45)	Low	Inhibition of cell growth	(46)
ERRγ (NR3B3)	High/low	Promotion or inhibition of cell growth	(36, 47, 48)	Low	Inhibition of cell growth	(49)

## ERRs in Breast Cancer

Breast cancer is the leading cause of cancer-related death in women worldwide (50). It is primarily a hormone-dependent disease that can be regulated by the status of steroid hormones such as estrogen and progesterone. Unfortunately, the emergence of hormone-resistant tumor cells after years of treatment is a major issue affecting patients with breast cancer (7). Breast cancers can be divided into five subtypes that vary in their treatment options and survival outcomes based on gene expression profiles (51–54). ERα positive (ERα+) and progesterone receptor positive (PR+) tumors account for approximately 70% of all cases (55, 56). These ERα+/PR+ tumors can be further classified into HER2+ and HER2− subtypes depending on *epidermal growth factor receptor 2*, *ErbB-2*, or *HER2* gene expression. Blocking the estrogen production or estrogen binding to the receptor by tamoxifen or aromatase inhibitors is the standard treatment for both early and advanced ERα+ breast cancer (57, 58). For ERα negative (ERα−), progesterone receptor negative (PR−), and HER2+ (ERα−/PR−/HER2+) breast tumors, a combination of pertuzumab, trastuzumab, and docetaxel has been effective (59). However, there are still no approved targeted therapies for triple negative ERα−/PR−/HER2−breast tumors (10–17% of all breast cancer cases) (60, 61), or the normal breast-like or basal-like cancer subtype (15% of the cases) (62, 63), which are mostly triple negative and frequently have *TP53* mutations (64).

Much attention has been paid to the role of ERRs in breast cancer, as they are orphan nuclear receptors closely related to ERs. ERRα expression in breast tumors is often high, and it is expressed in tumors with poor prognosis (36). In samples from various cohorts of patients with breast cancer, ERRα mRNA positively correlates with the expression of the oncogene *ERBB2* and inversely correlates to that of ERα and PR, which are considered as good prognostic markers for patients with breast cancer (36). The expression of ERRα mRNA and protein positively correlates with the coactivator amplified in breast cancer 1 (AIB1), also known as SRC-3 (65). However, ERRα is able to act as both a transcriptional activator and repressor depending on the cellular context, promoting or inhibiting tumor growth in breast cancer (42). In ER− breast cancer cells, ERRα functions as a transcriptional activator constitutively interacting with coactivators and binding to EREs independently of any ligands. Consequently, ERRα competes with ER in the regulation of estrogen-responsive genes such as the *estrogen-regulated trefoil factor 1 (TFF1)* (65, 66) and *vascular endothelial growth factor (VEGF)* (34, 43). In ER+ breast cancer cells, ERRα functions as a transcriptional repressor, interacting with corepressors and binding to negative EREs (42).

ERRα also plays a role in bone metastasis, which occurs in up to 70% of patients with advanced breast cancer (44). In a mouse xenograft model of metastatic human breast cancer, overexpression of wild-type ERRα-reduced metastasis and breast cancer cell growth in the bone, likely by upregulating the osteoclastogenesis inhibitor, osteoprotegerin (OPG). By contrast, ERRα overexpression increases breast cancer cell growth in the mammary gland and the expression of VEGF. Thus, ERRα plays dual roles, promoting the progression and invasion of primary tumors by decreasing osteolytic lesions in the bone (44).

It has been suggested that the induction of *c-myc* expression by estrogen occurs through the “non-classical” pathway – without binding of ERα to its promoter (67). Another study demonstrated a positive correlation between ERRα, c-myc, and aromatase (37), an enzyme that may enhance estrogen production and stimulate breast cancer progression (38). These studies propose that ERR could play an important role in alternative pathway to classical ERs-dependent pathway in cell signaling through aromatase and *c-myc*.

ERRα participates in the enhancement of estrogen production by the activation of steroid sulfotransferase (SULT2A1), which maintains high level of peripheral dehydroepiandrosterone sulfate (DHEAS), an important dehydroepiandrosterone (DHEA) metabolite in estrogen synthesis in certain tissues (68). It has also been reported that SULT2A1 inactivates tamoxifen and raloxifene (69). Thus, SULT2A1 activation by ERRα can partly explain the resistance of breast cancer cells expressing ERRα to SERM therapy.

A clinical study analyzing 102 breast cancer samples revealed that the expression of ERRα in more than 10% of malignant cells was associated with a 20% decrease in overall disease survival at 13 years (70). In this work, association between ERα and ERE-containing estrogen-responsive genes was markedly altered according to ERRα status in breast cancer tissues, suggesting that ERRα possibly modulates ERα-mediated and ERE-dependent transcription, and changes the expression of estrogen-responsive genes in breast cancer cells. In a previous clinical study, high levels of ERRα mRNA correlated with ERα-tumor status in 38 tumor specimens (36). These two studies suggest that ERRα mRNA and protein expression are associated with an unfavorable prognosis, and increase the risk of recurrence of breast cancer.

ERRγ also acts differently depending on ER expression. In breast tumors co-expressing ER and PR, ERRγ induces E-cadherin expression and promotes the mesenchymal-to-epithelial (MET) transition, resulting in the inhibition of tumor growth (36, 47). It has been shown that an AAAG tetranucleotide polymorphism in the untranslated region of the *ERR*γ gene is associated with breast cancer predisposition (71), and that ERRγ mediates tamoxifen resistance in invasive lobular breast cancer. In human breast cancer specimens, both ERRγ mRNA and protein expression are upregulated compared with normal samples, and exogenously transfected ERRγ increased breast cancer cell proliferation (36, 48). Tumors overexpressing ERRγ are also frequently steroid receptor positive, which may reflect hormonal sensitivity and a preferable clinical outcome. Thus, ERRγ mRNA expression is associated with a favorable prognosis of patients with breast cancer (36).

ERRβ expression has also been detected in breast tumors. It has been shown that ERRβ expression is associated with that of ERβ, and that ERRβ levels inversely correlate with the S-phase fraction, suggesting that this orphan receptor inhibits cellular proliferation, or possibly promotes cellular differentiation (36). However, another report found that it acts as a proliferative gene (45). Thus, the potential role of ERRβ in breast cancer remains unclear.

## ERRs in Prostate Cancer

Prostate cancer, which is dependent on androgens for proliferation and survival, is the second-leading cause of cancer-related mortality, after lung cancer, in men from developed countries (72). With early diagnosis, radical prostatectomy and/or radiation therapy are potentially curative. For advanced or metastatic prostate cancer, hormonal therapies, reducing androgen levels by surgical or chemical castration or inhibiting the AR protein by small molecules, are used (57). However, after an initial response, the cancer eventually recurs in an incurable, castration-resistant form (5, 6), as a result of amplification of AR protein, mutations of *AR* gene, and elevated production of AR variants (73).

In addition to androgen-signaling pathways, estrogen-signaling pathways are implicated in the development of prostate cancer (1), and estrogen has been used for the treatment of advanced prostate cancer (74). The direct effect of estrogens on normal and malignant prostate tissues is assumed to be mediated through ERα and ERβ (75, 76).

ERRα mRNA has been detected in prostate cancer cell lines and human prostate cancer tissue (77). Although a heterogeneous ERRα staining was found in immunohistochemical analysis using prostate cancer tissues with low Gleason score (GS), increased ERRα protein expression was detected in human prostate tissue from 106 surgical resected prostate samples in a study that showed a positive correlation between ERRα expression and GS (78). The enhanced expression of ERRα might play a role in the development of human prostate cancer and serve as a significant prognostic factor for the disease.

By contrast, reduced ERRβ and ERRγ expression in some prostatic carcinomas has been reported (77), and overexpression of ERRβ or ERRγ results in the suppression of cell proliferation in both androgen-sensitive and insensitive prostate cancer cells, suggesting that these receptors present antiproliferative or tumor-suppressing functions in prostate cancer (46, 49). Furthermore, it has been shown that ERRβ directly transactivates a promoter upstream of the cyclin-dependent kinase inhibitor, *p21* gene, resulting in the inhibition of the cell cycle progression (46). Clinicopathological studies for the expression of both orphan receptors in human prostate tissues have been performed using immunohistochemical analysis (79). Cancerous lesions and benign foci obtained from radical prostatectomy were stained and immunoreactivity scores (IR scores) were evaluated from the proportion of immunoreactive cells and staining intensity, revealing that ERRβ and ERRγ IR scores are significantly lower in cancerous tissues. In the same study, patients with high ERRα IR score, but low ERRγ IR score, presented a poorer cancer-specific survival when compared to the other group, suggesting that the expression of ERRβ and ERRγ could be a useful prognostic indicator of prostate cancer (79).

## ERRs Agonists and Antagonists

Although no endogenous ligands have been identified for these receptors several natural phytoestrogens (three isoflavones: genistein, daidzein, and biochanin A, and one flavone: 6, 3, 4-trihydroxyflavone) have been identified as potential ligands of these receptors with agonistic activities, by structure-based virtual screening and biological functional assays (80, 81). Phytoestrogens are produced by plants, and represent the major natural exogenous sources of estrogenic compounds. DY131 is another ERR agonist, specific to ERRβ and ERRγ that was shown to enhance growth inhibition, which was caused by overexpression of these nuclear receptors (46, 49). Inhibition of ERRα with the inverse agonist XCT790 reduces cell proliferation of various cancer cell lines, including prostate and breast cancer cells (39).

On the other hand, diethylstilbestrol (DB00255) and the tamoxifen metabolite, 4-hydroxytamoxifen, have been shown to interact with ERRβ and ERRγ and act as antagonists (82, 83). SR16388, a novel steroidal antiestrogen, inhibits the interaction between its coactivator peroxisome proliferator-activated receptor γ coactivator-1 α to inhibit ERR α activity (84).

## Conclusion

Although the role of orphan nuclear receptors in cancer is becoming clearer as a result of advances in previous studies, their function still remains to be elucidated. A better understanding of ERRs in breast and prostate cancer will provide new insights into cancer biology as well as the discovery of novel small molecules that bind to these orphan receptors. This knowledge will be helpful for the identification of novel hormonal therapeutic strategies and cancer treatments.

## Conflict of Interest Statement

The authors declare that the research was conducted in the absence of any commercial or financial relationships that could be construed as a potential conflict of interest.
